# A mindful approach to controlling intrusive thoughts

**DOI:** 10.1038/s41598-023-37447-9

**Published:** 2023-07-06

**Authors:** S. M. Ashton, A. Sambeth, C. W. E. M. Quaedflieg

**Affiliations:** Department of Neuropsychology and Psychopharmacology, Maastricht University, Maastricht, The Netherlands

**Keywords:** Cognitive control, Forgetting

## Abstract

**Abstract:**

Intrusive thoughts of negative experiences can pose a threat to our well-being. To some extent, unwanted memories can be intentionally controlled via an executive control mechanism that downregulates the occurrence of intrusions. Mindfulness training can improve executive control. It is not known whether mindfulness training can be used as an intervention to improve intentional memory control and reduce intrusions. To this end, 148 healthy participants completed a 10-day app-based mindfulness training or an active control task. At baseline, inhibitory control and working memory were assessed as measures of executive functioning. Post-mindfulness training, intrusions were assessed via the Think/No-Think task. It was expected that mindfulness training would reduce intrusions. Furthermore, we hypothesised that this would be moderated by baseline executive functioning. Results revealed that, contrary to our hypothesis, both groups increased equally in dispositional mindfulness between baseline and post-test. As such, our exploratory analysis revealed that higher dispositional mindfulness across both groups resulted in fewer intrusions and enhanced the ability to downregulate intrusions over time. Furthermore, this effect was moderated by inhibitory control at baseline. These results provide insight into factors that can improve the ability to control unwanted memories, which could have considerable implications for treatments in psychopathologies characterized by the frequent occurrence of intrusive thoughts.

**Protocol registration:**

The stage 1 protocol for this Registered Report was accepted in principle on 11th March, 2022. The protocol, as accepted by the journal, can be found at: 10.17605/OSF.IO/U8SJN.

## Introduction

Intentional memory control serves as an adaptive emotion regulation strategy, allowing us to suppress unwanted thoughts of negative experiences that pose a threat to our well-being^1–3^. Psychopathologies that are characterized by the occurrence of intrusive thoughts, such as post-traumatic stress disorder (PTSD), show a deficit in intentional memory control (for meta-analysis, see^4^). In the case that suppression fails, unwanted thoughts involuntarily purge into awareness^5^. In the context of intentional memory control, these counter-intentional memory retrievals are classified as intrusions.

One method to investigate intentional memory control is via the Think/No-Think paradigm (T/NT)^6^. Participants first learn associations between cue-target pairs (in the present study, neutral image pairs). After learning, participants perform the T/NT phase in which they must either actively retrieve the target in response to the cue (Think trials), or suppress thoughts of the target (No-Think trials). In the final part of the T/NT procedure, participants undergo a final memory test for all cue-target pairs learned during the first task. In general, recall for No-Think items tends to be reduced when compared to recall for baseline items (not cued in the T/NT phase). To intentionally suppress memory retrieval, the executive control network engages the right dorsolateral prefrontal cortex (dlPFC), down-regulating activity in the hippocampus^7–9^. This down-regulation is enhanced when an intrusion occurs, allowing it to be purged from awareness and inhibiting memory retrieval^10,11^. In addition, intrusion control has been found to correlate with later forgetting of these memories, in that those who demonstrate a steeper decline in intrusions show an increased forgetting effect^5,10^.

Executive control is suggested to be a key mechanism to regulate the retrieval of unwanted memories^6,12^. One method of training that has been found to improve executive control is the practice of mindfulness (for review, see^13^). Mindfulness training (MT) can be taught in a variety of forms, although the key concept is grounded in the ability to focus attention on the present moment^14^. The benefits extend beyond the well-established positive effects on psychological well-being (for meta-analysis, see^15^) and further translate into enhanced executive control. MT has been found to improve executive functioning in healthy novices^16–20^. The effect sizes do vary between executive functions (for meta-analysis, see^21^), with the most change observed for inhibitory control (for review, see^13^). The discussed studies demonstrate these changes after longer-term interventions (e.g. 6–8 weeks). However, enhanced cognitive control has also been observed following a single app-based session of mindfulness meditation^22^. Although, independent of duration, null effects using app-based interventions have also been observed (e.g.^23^). In addition, in the absence of an intervention, experienced meditators have been found to have enhanced inhibition compared to non-meditators, which also correlated with self-reported mindfulness^24^. At a neural level, MT has been found to enhance activity in brain regions associated with intentional memory control. MT has been found to increase dlPFC activity during executive processing, consequently increasing recruitment of the top-down mechanisms needed for executive control^16^. These shared neural correlates indicate that MT may enhance the ability to intentionally control memories and reduce intrusions.

Individual differences in executive control may account for some variability in intentional memory control, which could explain why some individuals fail to suppress and experience intrusions^25^. Two constructs that are considered to be core executive control functions are inhibitory control and working memory (for review, see^26^). A higher working memory capacity, as measured by the Operation Span task (OSPAN; see^27^), has been associated with experiencing fewer intrusions during a thought suppression task^28–30^. During intentional memory control (i.e. No-Think trials), inhibition of the target memory is engaged, which allows for selective retrieval of relevant information^25^. This same mechanism underlies other measures of inhibition, such as the Stroop test (see^27^). Alternative theories of intentional memory control suggest that suppression-induced forgetting can be better explained by interference, rather than inhibition^31^. This difference could result from different strategies employed to suppress unwanted content. The current study will instruct participants to use direct suppression as a strategy based on its relation to inhibitory control^32,33^. This strategy will be employed to investigate intentional control of memory intrusions.

The aim of the current study is to investigate whether mindfulness training can be used to enhance intrusion control, and whether this is moderated by executive control functions. The study employed a 10 day app-based intervention that has shown to be effective in increasing dispositional mindfulness^34,35^. We employed an adapted version of the T/NT paradigm to measure the course of intrusions experienced during intentional suppression^2,5,10,11,36^. On a trial-by-trial basis, participants reported to what extent the target came to mind during presentation of the cue. Using this paradigm, previous research has established that the occurrence of unwanted memory intrusions decrease over repeated suppression (i.e. No-Think trials^5,10,11,37^). In the current study, measures of intrusion control were compared between the mindfulness and active control group.

### Hypotheses (see Table 1)

**Table 1:** Design table.

Question	Hypothesis (if applicable)	Sampling plan (e.g. power analysis)	Analysis plan	Interpretation given to different outcomes
Does mindfulness training influence the ability to intentionally suppress intrusions and is this effect moderated by baseline executive functioning	H1: MT training will enhance suppression of intrusions (i.e. fewer total intrusions and more negative Index of Intrusion Control (IIC)) in comparison to the control condition. This effect will be moderated by baseline executive functioning (i.e. decreased scores on the Stroop and increased scores on the OSPAN will result in a greater enhancement in intrusion control)	The *a-priori* power calculation using G*Power (*f*^*2*^ = *0.15, *α = 0.025, 1 − β = 0.95) indicates a sample size of *N* = 136	Two moderation analyses will be used to test the direct effect of MT on intrusions (total number of intrusions, IIC) and its moderation by executive functioning (2 moderator variables: Stroop and OSPAN scores) These analyses will be supplemented with corresponding Bayesian regression models using a Jeffreys-Zellner-Siow (JZS) prior distribution (scale 0.354), reporting Bayes factor	In the case of significant differences between groups in compliance scores, subjective suppression ability or psychological characteristics (BDI-II; ETISR-SF; RRS-10; STAI-T; TICS), these scores will be added as a covariate to the moderation analyses

H1: We hypothesised that MT training would enhance intrusion control in comparison to the control condition. Furthermore, this effect would be moderated by baseline executive functioning (i.e. increased executive functioning results in a greater enhancement in intrusion control).

## Methods

### Ethics information

The test protocols were approved by the ethics committee of the Faculty of Psychology and Neuroscience, Maastricht University and were conducted in accordance with the provisions of the World Medical Association Declaration of Helsinki (reference: OZL_187_02_01_2018_S4). All participants provided informed consent. Participants were reimbursed with university credits or were entered into a lottery. Four winners were randomly selected from the lottery and compensated with 25 euros. Our advertisement stated that our study aimed to investigates the relationship between mindfulness and attention.

### Design

**Figure 1:**
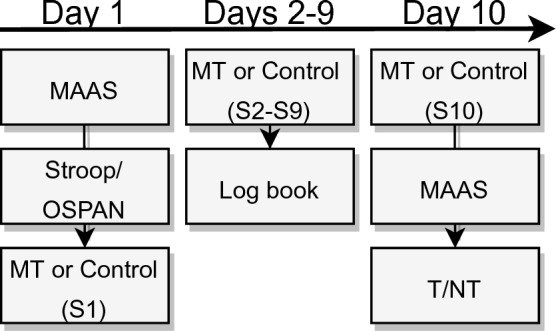
Outline of the study procedure. On day 1, participants completed the Mindfulness Attention Awareness Scale (MAAS), followed by measures of executive functioning (Stroop and Operation span task (OSPAN), counterbalanced in order) then concluded with the first mindfulness training (MT) or control task (S1 = Session 1). On days 2–9, participants completed daily sessions of MT or the control task (S2 = Session 2–S9 = Session 9). On day 10, participants began with their final session of the MT or control task (S10 = Session 10) and completed the post-training measures of MAAS and the 3 phases of the intentional memory control task (T/NT).

The study ran over 10 consecutive days, consisting of two lab sessions, one on day 1 and one on day 10, and online sessions on days 2–9 (see Fig. 1). The first lab session ran for approximately 45 min and the final lab session ran for approximately 1.5 h. The labs sessions were scheduled at the same time of day (either morning or afternoon) for each participant. The online sessions ran for approximately 10 min each day. The sample was pseudo-randomly divided into two equal groups: the mindfulness training group (MT) and the control group. The time of testing and type of compensation were equally distributed between the groups (see Table 2). All participants were instructed not to take part in any other mindfulness task or practice, other than what they had been assigned. To ensure blinding during data collection, different experimenters performed test days 1 and 10. The experimenter on day 1 explained the procedure for days 2–9 to the participant. The second experimenter on day 10 was not aware of the condition. To ensure blinding during analysis, an independent researcher recoded the conditions after data collection.

### Materials

#### Mindfulness questionnaire

The Mindfulness Attention Awareness Scale (MAAS)^38^ contains a list of 15 statements designed to assess core characteristics of mindfulness. For example: “I find it difficult to stay focused on what’s happening in the present”. Participants were asked to rate how frequently each of the statements applied to their everyday experience. Each item was scored on a 6 point scale from 1 (almost always) to 6 (almost never). The responses were then summed, with higher scores indicating increased mindfulness. The MAAS was administered at baseline and post training.

In addition, participants were asked about their attitude towards mindfulness training at the start of the experiment. Participants were asked the statement: “Mindfulness has the potential to be beneficial” and responded on a likert scale response from 1 (strongly disagree) to 5 (strongly agree). This was used as a single score to gain a quantifiable measure of attitudes toward mindfulness^35^.

#### Mindfulness training condition

Mindfulness training was delivered via the Headspace smartphone app (http://www.headspace.com). Participants were instructed to complete the course ‘Basics’, which gave 10 introductory sessions that consisted of daily mindfulness meditation exercises with a duration of approximately 5 min each. These sessions are tailored to individuals who are naïve to mindfulness meditation and aim to teach the basic techniques of the practice, such as breath awareness and body scanning^12^. The first and last sessions were completed in the lab, whereas sessions 2–9 were completed online. To check adherence, participants were asked to complete a logbook entry after each session. Participants were asked to note which session they had completed and write a brief reflection on their experience in two or more sentences. Reminders were sent each day via email with instructions and links to the study materials.

#### Control condition

Participants in the control group were given a playlist containing 10 podcasts from ‘Radio Headspace’ (http://www.headspace.com/podcast), with approximately the same duration as the sessions for the MT condition (i.e. 5 min each). The content of the podcasts relate to the benefits of mindfulness meditation and promote positive well-being, but do not entail direct practicing of MT techniques. As such, the control task was based on mindfulness knowledge, rather than on mindfulness practice. This aimed to ensures that any effects can be attributed to mindfulness practice specifically, rather than only a belief in the effects of mindfulness (see^39,40^ for methodological considerations for control groups in mindfulness research). To exclude the possibility of order effects, the order of the podcasts was randomized for each participant. To check adherence, participants were asked to complete a logbook entry after each session. Participants were asked to note which session they had completed and write a summary of the content in two or more sentences. The timings of the sessions and reminders matched those of the MT condition.

### Validity of the control task: pilot data

A pilot study was conducted to test the validity of the control task. Twenty-two participants were randomly allocated into either the MT or control group and asked to complete the corresponding 10-day program. Prior to the first session and following the final session, participants completed the MAAS. To check adherence, participants were asked to note which session they had completed via a daily logbook. All participants completed ≥ 8 sessions.

To determine changes in mindfulness, we used a 2 × 2 ANOVA with condition (2 levels: MT vs. control) as the between subjects factor and time (2 levels: baseline vs. post-training) as the within subjects factor. We supplemented this analysis using a Bayesian repeated-measures ANOVA compared to the null model (including subjects and main effects). Results showed a significant interaction effect (Time*Condition: *F*_(1,20)_ = 5.75, *p* = 0.026, η_p_^2^ = 0.22, BF^10^ = 2.43). Follow up within subjects analyses showed that the MAAS scores did not change between baseline and post-training for the control group (*t*_(10)_ = − 1.65, *p* = 0.13, BF^01^ = 1.18), but did increase significantly between time points for the MT group (*t*_(10)_ = 4.17, *p* = 0.002, BF^10^ = 23.45). There was no significant difference between the groups at baseline (t_(20)_ = 0.86, *p* = 0.40, BF^01^ = 2.00).

#### Stroop Test

The Stroop Test^41^ was used to assess inhibitory control. The task cued names of colours, written in coloured text. Participants responded by naming the colour of the word rather than the meaning of the word. The task cued 3 trial types: congruent, incongruent and control. The words were written in either the same colour as the word (congruent trials) or in a different colour than the word (incongruent trials). Control trials presented coloured blocks. Participants responded by indicating the colour of the word or block by pushing the corresponding button. The task consisted of 4 colours (red, green, blue and black). Each colour was repeated 7 times within each stimulus type (congruent, incongruent and control). This resulted in 84 trials in total, with an inter-trial interval of 200 ms. The trials were shown in a random order. The font size was set to 8% of the screen height (corresponding to font size 74 for the current set up). The task duration was approximately 2 min. We calculated an interference score, based on the reaction time of correct responses for incongruent, congruent and control trials (Incongruent − ((Control + Congruent)/2))^42^. The scores were then multiplied by − 1, so that higher scores are indicative of decreased interference, reflecting increased inhibitory control.

#### Operation span task

The OSPAN^43^ was used to assess working memory. Participants were presented with a sequence of letters, ranging from 3 to 7 in length. Each letter in the sequence was followed by a mathematic equation, followed by a potential solution to the equation. Participants indicated whether the solution was correct or incorrect. At the end of the sequence, participants indicated the letters seen between each maths equation via a letter matrix. The task started with a short practice phase of 4 trials, followed by a test session of 15 trials. The inter-trial interval was 500 ms. The total duration was approximately 20 min. Each letter in the sequence was shown for 1 s. The length of maths trials were relative to the participant’s performance on the practice phase, taking the mean response time from the practice trials and adding 2.5 times the standard deviation for the test session. We adopted the absolute scoring method, which calculates the sum score of all letter sequences recalled with 100% accuracy.

#### Measures of intentional memory control: Think/No-Think paradigm

**Figure 2:**
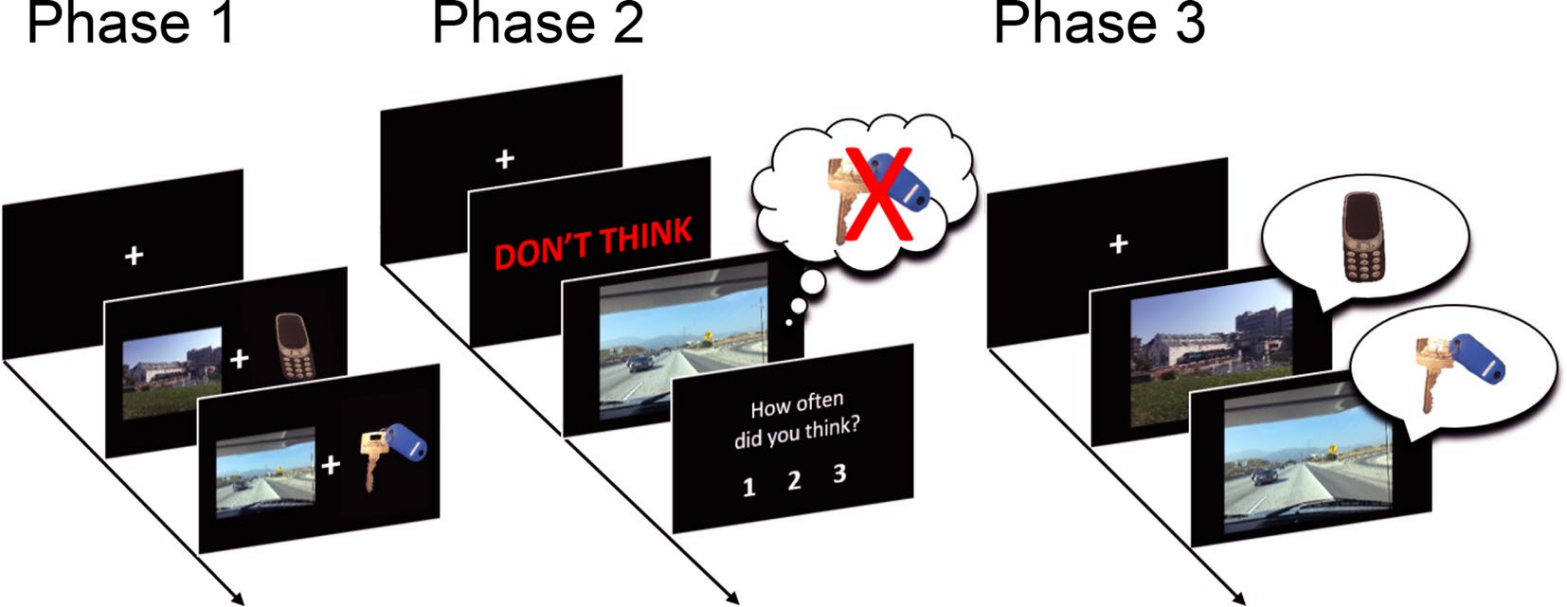
Outline of the T/NT task. In phase 1, participants memorised cue-target pairs and thereafter recalled the target in response to the cue. In phase 2, participants performed the T/NT task. An example of a No-Think (NT) trial is shown. Participants were first presented with the instruction (Think or Don’t Think), followed by a cue. For NT trials, participants were instructed to suppress thoughts of the target whilst the cue is on the screen. At the end of each trial, participants reported how often they had thought of the target (1 = never; 2 = briefly; 3 = often). Responses of 2 or 3 on NT trials were used to determine intrusions. Participants then completed the final recall test and recalled all targets in response to each cue. Correctly recalled cue-target pairs were calculated for each stimulus type (Think, No-Think and baseline) and used to calculate the suppression-induced forgetting index.

Intentional memory control was assessed via an adapted version of the Think/No-Think paradigm (T/NT)^6^. The task comprised 3 phases: learning; the Think/No-Think task; and the final recall test (see Fig. 2).

#### Stimuli for the T/NT paradigm

The stimuli used for the cue-target pairs comprised neutrally rated images selected from 3 picture databases (GAPED^44^; IAPS^45^; NAPS^46^). The cues were images of scenes/landscapes and the targets were images of objects. Images of objects were edited to delete their backgrounds and centre the objects in the frame (Photoshop; Adobe, San Jose, CA, USA). Thirty-six cue-target pairs were formed, consisting of 72 images (Valence ratings: cues: *M* = 5.17, *SD* = 0.40; targets: *M* = 5.35, *SD* = 0.43).

Allocation of the cue-target pairs were randomized for each participant, with the restriction that all pairs contained unrelated image combinations (i.e. the subject of the images do not share a similar meaning or purpose). Based on an independent sample (*N* = 10), a list of labels (i.e. names for the target objects) were created to develop a scoring template for the T/NT task. As the naming of the objects is subjective, the template was used as a guideline. As such, the final learning round and the final recall test were voice recorded to allow for an additional independent rater to score each item. The raters were instructed that self-correction during these rounds should be considered correct (i.e. the final answer given in the allotted time). For the final learning round, the inter-rater reliability was near perfect (*κ* = 0.999), with the 3 items with disagreement removed from all analyses. For the recall test, the inter-rater reliability was also near perfect (*κ* = 0.998), with the 4 items with disagreement removed from analyses.

#### Phase 1

The learning phase tasked participants to memorise 36 cue-target pairs. The cue-target pairs were presented consecutively for 4 s each. This procedure was repeated twice. This was followed by 3 cued-recall tests. Each cue was presented consecutively, during which time the participant recalled the corresponding target aloud. Each cue appeared on the screen for 10 s. If the participant recalled the target before 10 s, they could proceed to the next trial. For the first 2 cued-recall tests, the corresponding target was then shown as feedback for 4 s. In the final cued-recall test, participants were shown each cue once again for 4 s each. Participants recalled the corresponding target aloud and the experimenter scored the responses. For this test, no feedback was shown. Eleven cue-target pairs were randomly selected for each of the stimulus types (Think, No-Think and baseline). The 3 remaining cue-target pairs were used as practice items. Participants who did not meet the learning criteria of ≥ 70% (excluding practice items, i.e. 24/33 of the cue-target pairs) were excluded (*n* = 11), and the procedure stopped at this point. Only items learned during the final learning round of the T/NT task were used for subsequent analyses.

#### Phase 2

The T/NT task was framed to participants as an attention task. This phase presented 11 Think cues and 11 No-Think cues. Each cue was repeated 10 times in a random order, with the restriction that no more than 2 of the same stimulus type could be shown consecutively. This resulted in 220 trials in total. The trials were split over five blocks that were separated by 45 s breaks, with 2 repetitions of each cue in each block. Each trial began with a centred fixation cross for 1.5 s, followed by an instruction (either ‘Think’ or ‘Don’t Think’, written in green and red text, respectively) for 1 s, followed by a corresponding cue presented for 4 s. During Think trials, participants were instructed to retrieve the memory of the associated target during presentation of the cue. To ensure adherence to the task instructions, participants were asked to state the name of the target aloud. During No-Think trials, participants were instructed to block out thoughts of the associated target. If the target did come to mind, they were instructed to actively push the thought out of awareness. Participants were instructed to do so without using distraction tactics, to maintain their focus on the cue and to complete the task in silence (see materials on OSF for participant instructions). At the end of each trial, participants were shown the question “How often did you think about the target?”, and responded by pressing either 1, 2 or 3 on their keyboard (1 = never; 2 = briefly; 3 = often). Participants had a response window of 3 s to provide this rating, although they were instructed to provide the response as quickly and as accurately as possible. If the participant responded before the 3 s, a black screen was presented until the next trial began. Therefore, each trial during phase 2 was the same length (9.5 s). Responses recorded during No-Think trials were used to determine intrusions (cf. analysis: pre-processing steps).

Participants completed a short practice session before the main T/NT task. The practice session consisted of 3 blocks, with 1 Think trial and 2 No-Think trials in each. The experimenter asked participants to verbally discuss their strategy after each block in order to check understanding and adherence to the task.

#### Phase 3

In the final recall test, all 33 Think, No-Think and baseline items were presented. Each cue was presented for 4 s, during which time participants recalled the corresponding target aloud. The experimenter scored the responses and the number of correctly recalled cue-target pairs were calculated for each stimulus type (Think, No-Think and baseline) and used to calculate the suppression induced forgetting (SIF) index (cf. analysis: pre-processing steps). The protocol registration stated that participants would complete a short practice session before this phase, presenting the 3 practice items. These trials were not included, as they were accidentally overlooked when programming the task script.

#### Subjective suppression ability

At the end of the T/NT task, participants completed a 3 item questionnaire that assessed participants’ subjective ability during No-Think trials: (1) How motivated were you to block-out the red “Don’t Think” targets (2) How difficult did you find it to block-out the “Don’t Think” target; (3) How well did you manage to block-out the “Don’t Think” target?”. Participants recorded their responses on a visual analogue scale (VAS) from 1 (not at all) to 100 (very) for each item. Scores on item 2 were reversed before taking the average of the 3 items. Higher scores are indicative of increased subjective suppression ability (see Table 2).

**Table 2:** Demographic and psychological characteristics of the sample, measures of mindfulness attitude, task compliance, subjective suppression ability and counterbalanced measures for time of testing and type of compensation.

			MT (*n* = 73)		Control (*n* = 75)		*t*	*p*	*d*
			*M*	*SD*	*M*	*SD*			
Age			20.10	1.95	20.69	2.71	1.54	0.13	0.25
BDI-II			11.97	9.49	11.27	6.18	0.54	0.59	0.087
ETISR-SF			2.12	1.44	2.36	1.67	0.94	0.35	0.15
RRS-10			23.63	4.61	22.89	4.80	0.95	0.34	0.16
STAI-T			45.85	8.91	46.03	8.78	0.12	0.90	0.02
TICS			13.01	6.24	13.64	5.21	0.66	0.51	0.11
Mindfulness attitude			4.34	0.87	4.35	0.71	0.032	0.97	0.013
Subjective suppression ability			58.26	15.44	54.17	15.44	1.71	0.090	0.28
Task compliance			1.16	1.03	1.52	1.01	2.13	0.035*	0.35

	MT			Control			*X*^*2*^	*p*	*ω*
Time of testing	a.m.	p.m.		a.m.	p.m.				
	35	38		38	37		0.110	0.74	0.027
Compensation	*Credit*	*Lottery*		*Credit*	*Lottery*				
	68	6		68	7		0.306	0.58	0.045
Gender	*M*	*F*	*N-B*	*M*	*F*	*N-B*			
	12	61	0	12	63	0	0.005	0.94	0.006

Significant differences between groups are indicated (*).

MT: Mindfulness training group; BDI-II: Becks Depression Inventory; ETISR-SF: Early Trauma Inventory Self-report (short version); RRS-10: Ruminative Response Scale; STAI-T: State-Trait Anxiety Inventory; TICS: Trier Inventory for Chronic Stress; Time of testing: a.m.: morning, p.m.: afternoon; Gender: M: male, F: female, N-B: non-binary.

We also asked additional diagnostic questions to check compliance with the suppression instructions during No-Think trials: (1) When I saw a red cue, I quickly checked to see whether I remembered the target; (2) After a red cue went off the screen, I checked to see if I still remembered the target; (3) When I saw a red cue, I thought about the target that went with it to purposely improve my memory for that pair (see^47^). Participants rated each of the 3 questions on a 5 point likert scale from 0 (never) to 4 (always). Participants were asked to respond 1 if the behaviour occurred by accident, and 2, 3 or 4 if done on purpose. This differentiation was added to add clarity on the meaning behind ‘intentionally’. Sum scores were calculated to gain a measure of compliance (see Table 2). Following the recommendations of Liu et al.^47^, participants with scores > 3 were excluded from analysis.

#### Procedure

During the baseline session (day 1), participants were first provided with information about the study before providing written consent. Participants then provided demographic information, followed by questionnaires measuring psychological characteristics and concluding with the MAAS. Participants then performed the Stroop and OSPAN tasks. The order of these tasks was counterbalanced. All tasks were presented via Inquisit 6 on a 17-in. LCD monitor. Participants were seated 60 cm away from the screen. Next, participants in the MT group completed their first mindfulness session and participants in the control group listened to their first podcast. All participants completed their logbook immediately following the session.

Days 2–9 were completed online. Participants completed their daily MT or podcast session and completed their logbook entry immediately following. Participants were advised to complete the session at a similar time each day at a time where they are able to focus.

During the post-training session (day 10), participants first completed either the final MT or podcast session and then completed the MAAS. This was then followed by the T/NT task. Lastly, participants were debriefed and compensated for their time (see Fig. 1).

### Sampling method

The *a*-*priori* power calculation using G*Power v3.1. (multiple regression model using 3 predictors: *f*^2^ = 0.15, α = 0.025, 1 − β = 0.95)^48^ indicated a sample size of *N* = 136 to detect the effects of MT on intrusion control and its moderation by executive functioning. The alpha level was adjusted via the Bonferroni correction to account for multiple testing on the two dependent variables to measure intrusions (total number of intrusions and Index of Intrusion control (IIC)^49^; cf. analysis: pre-processing steps). Note, we recruited until the included sample reached 75 participants for each group (10% over the minimum indicated by the power calculation) to allow for further exclusion after checking the data or removal of potential outliers.

The study recruited 214 psychologically healthy participants aged 18–35. Inclusion of participants was checked via an online pre-screening and assessed the following criteria: little to no experience with mindfulness meditation in the past 6 months (≤ weekly practice); no current or past psychiatric condition within the past 3 years; no neurological disorder; have not participated in another study by our research group investigating intentional memory control (two participants were excluded during testing for this criteria). Participants who failed to complete > 1 online sessions were excluded from the sample (*n* = 17). To ensure a high response rate for the intrusion control measures, participants were excluded if they had 10% missing intrusion trials (i.e. > 2) in any one of the 10 blocks of the T/NT task (*n* = 1). In addition, participants with scores > 3 on the compliance questionnaire following the T/NT procedure were excluded from the sample (*n* = 24). Moreover, 6 participants were excluded as they did not attend the second lab session and 5 participants were excluded due to experimenter error. The data were replaced until the required sample size was met. A final sample of *N* = 148 remained for analysis (MT: *n* = 73, control: *n* = 75). To gain insight into the characteristics of our sample, we collected demographic data on age, gender and data on psychological characteristics (Becks Depression Inventory: BDI-II; Early Trauma Inventory Self-report (short version): ETISR-SF; Ruminative Response Scale: RRS-10; State-Trait Anxiety Inventory: STAI-T; Trier Inventory for Chronic Stress: TICS), presented in Table 2. In the case of significant group differences, scores from the related questionnaire were added as a covariate to the main analysis.

### Analysis

#### Pre-processing steps

As our research question focused on intentional suppression, our analysis focused on No-Think trials. Responses recorded during No-Think trials were used to determine intrusions. In line with previous studies, responses of 2 (briefly) or 3 (often) were classified as an intrusion^5,10,36^. The frequency of intrusions were calculated for each block of the T/NT task, taking into account initial learning of No-Think items and the number of valid trials (i.e. removing missing responses; *M* = 0.41%). From these responses, we calculated 2 measures of intrusions: (1) the total frequency of intrusions across the 10 blocks of the T/NT task and (2) Index of Intrusion Control (IIC) reflecting the change in the frequency of intrusions across the 10 blocks^49^. Increasingly negative values indicate a larger decrease in intrusions over time.

The SIF index was calculated by subtracting the recall of No-Think items from baseline items and then dividing by baseline items (((baseline – No-Think)/baseline); see^10,50^). This results in a subject-specific measure of forgetting relative to baseline memory performance, with higher positive index scores indicating increased forgetting. Two participants were removed from this analysis due to no audio recording from experimenter error, therefore independent scoring could not be determined.

The data were screened prior to analysis to check if the assumptions for each statistical model were met. The predictor variables for the moderation analyses were checked for multivariate outliers using Cook’s distance. For consistency, each section of the results first presents the sample included for the main moderation analysis (MT; *n* = 73; Control; *n* = 75). The preregistered protocol states that outliers for univariate analyses would be determined on the related dependent variables using the interquartile range method (using 1.5 as the scale). For transparency, we also present the results of each univariate analysis without identified outliers or justify why this approach is no longer fitting for the data in reality.

#### Preliminary analysis

First, we checked whether the mindfulness training was effective. To determine training-induced changes in mindfulness, we used a 2 × 2 ANOVA on scores from the MAAS with condition (2 levels: MT vs. control) as the between subjects factor and time (2 levels: baseline vs. post-training) as the within subjects factor. As an additional control measure, participants’ attitude toward mindfulness was compared between the groups using an independent samples *t*-test. In the case of a significant group difference, mindfulness attitude scores would be added as a covariate to the ANOVA measuring the effectiveness of mindfulness training.

To check the cue-target pairs were learned to the same extent between the groups, we performed an independent samples *t*-test on the final learning scores of the T/NT task. As a manipulation check for the T/NT task, the SIF index was assessed for the total sample after exclusions using a one sample *t*-test to determine whether the value differs from a test value of 0.

To analyse additional control measures, compliance scores and subjective suppression ability were compared between the groups using an independent samples *t*-test. In the case of significant group differences, these scores were added as a covariate to the moderation model assessing intrusion control.

All findings are supplemented with a corresponding Bayesian analysis. Bayes factors are reported for univariate analyses using a Cauchy prior distribution (scale √2/2) and Cauchy prior distribution (r scale 0.5) for multivariate analyses and compared to the null model (including subjects and main effects).

#### Analysis plan per hypothesis

Moderation analyses using the PROCESS tool for SPSS (Model 2 with 1000 bootstrapping) were used to test the direct effect of MT on intrusion control (total number of intrusions; IIC) and its moderation by executive functioning (2 variables: Stroop and OSPAN scores). Findings were supplemented with corresponding Bayesian regression models using a Jeffreys-Zellner-Siow (JZS) prior distribution (scale 0.354).

## Results

### Mindfulness training and active control task both increased dispositional mindfulness

First, participants’ attitude toward mindfulness was compared between the groups using an independent samples *t*-test. No difference was observed between the groups (*t*_(146)_ = 0.032, *p* = 0.97, *d* = 0.005, BF^01^ = 5.65). The results do not change after removal of 4 outliers (*t*_(142)_ = − 0.80, *p* = 0.43, *d* = 0.13, BF^01^ = 4.18).

**Figure 3:**
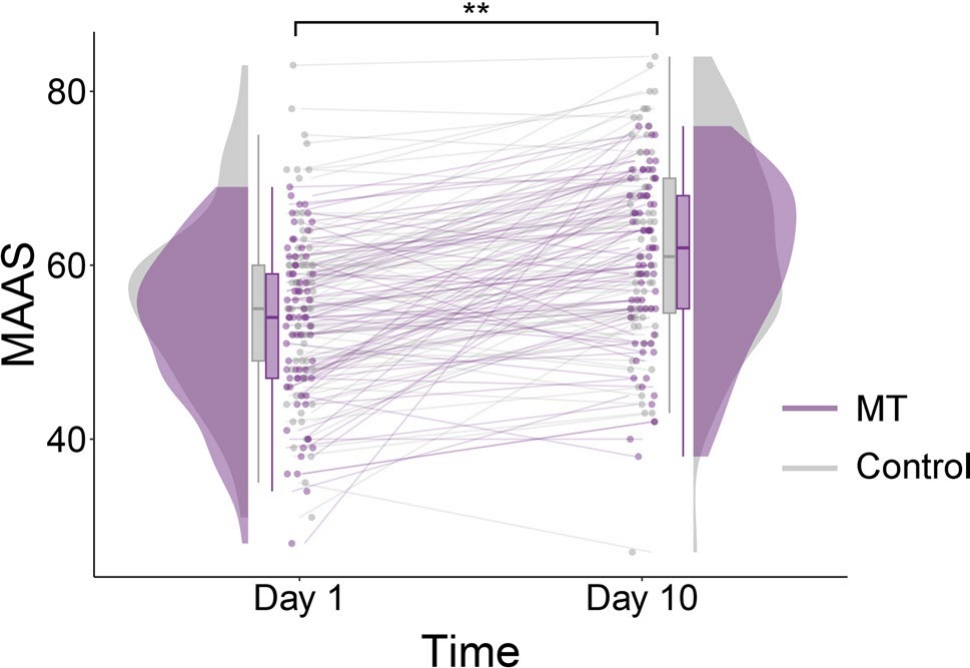
Participants in both groups increased in dispositional mindfulness. The plot displays a significant increase in dispositional mindfulness between day 1 and day 10 for both the Mindful (MT) and Control groups. The raincloud plots^53^ display the mean values within boxplots, as well as individual data points for each time point and the distribution for both groups at each time point.

Next, we checked whether the mindfulness training was effective. To determine training-induced changes in dispositional mindfulness, we used a 2 × 2 ANOVA on scores from the MAAS with group (2 levels: MT vs. control) as the between subjects factor and time (2 levels: baseline vs. post-training) as the within subjects factor. Dispositional mindfulness increased between baseline and post-training (Time: *F*_(1,146)_ = 88.23, *p* < 0.001, η_p_^2^ = 0.38, BF^10^ > 100), and this effect was equal for both groups (Time*Group: *F*_(1,143)_ = 0.48, *p* = 0.49, η_p_^2^ = 0.003, BF^01^ = 4.51; see Fig. 3). There was no main effect of group (*F*_(1,146)_ = 2.02, *p* = 0.16, η_p_^2^ = 0.014, BF^01^ = 2.13). The results do not change when 9 outliers are removed from the analysis (Time: *F*_(1,137)_ = 100.99, *p* < 0.001, η_p_^2^ = 0.42, BF^10^ > 100; Time*Group: *F*_(1,137)_ = 0.46, *p* = 0.50, η_p_^2^ = 0.003, BF^01^ = 4.07; Group: *F*_(1,137)_ = 1.71, *p* = 0.19, η_p_^2^ = 0.012, BF^01^ = 2.05).

### High learning rates for the cue-target pairs in the Think/No-Think task

Overall, participants learned the cue-target pairs well (*M* = 97.31, *SD* = 5.48, range: 72.73–100) and learning did not differ between Think, No-Think and baseline stimulus types (*F*_(2, 294)_ = 0.010, *p* = 0.99, η_p_^2^ > 0.001, BF^01^ = 40.12). To check the cue-target pairs were learned to the same extent between the groups, we performed an independent samples *t*-test on the final learning scores of the T/NT task. Performance was comparable between groups (*t*_(146)_ = 0.067, *p* = 0.95, *d* = 0.011, BF^01^ = 5.64). Our preregistered approach for univariate analyses specified to remove outliers using the IQR method. As the mean learning was close to 100%, participants with scores < 95% were identified as outliers (*n* = 18). These ceiling effects render the IQR as an ineffective approach. In addition, the scores are within the expected range due to the learning threshold of 75%, therefore these cases are included in the analysis.

### Suppression-induced forgetting effect present in final recall task

As a manipulation check for the T/NT task, the SIF index was assessed for the total sample using a one-sample *t*-test. Results showed that the SIF index differed significantly from the test value 0 (*t*_(145)_ = 2.23, *p* = 0.027, *d* = 0.18, BF^10^ = 1.01), with a positive mean (*M* = 0.011, *SD* = 0.057) demonstrating an increased forgetting effect (i.e. impaired recall for No-Think items compared to baseline). Due to the high learning scores, the majority of the sample demonstrated recall scores of 100% (*n* = 125). Using the IQR, as specified in the preregistered report, all participants with scores less than 100% were identified as outliers (No-Think recall range: 60–100%; Baseline recall range: 81.82–100%). These ceiling effects for the final recall are in line with several previous studies^49,51,52^ and render the IQR as an ineffective approach. Therefore, we do not identify these cases as outliers and their data is included in the analysis.

### Participants who practiced mindfulness showed higher compliance to task instructions

To analyse additional control measures, compliance scores and subjective suppression ability were compared between the groups using an independent samples *t*-test. No difference was observed between groups for subjective suppression ability (*t*_(146)_ = 1.71, *p* = 0.090, *d* = 0.28, BF^01^ = 1.49). This result does not change after removing 2 outliers (*t*_(144)_ = 1.75, *p* = 0.082, *d* = 0.29, BF^01^ = 1.38).

Participants in the MT group showed significantly higher compliance compared to the control group (*t*_(146)_ = 2.13, *p* = 0.035, *d* = 0.35, BF^10^ = 1.39). As compliance has been suggested to influence intentional suppression^47^, compliance scores were added as a covariate to the moderation models assessing the primary hypothesis.

### Mindfulness practice and executive control as predictors of intrusion control

The overall models assessing the direct effect of MT on intrusions did not prove significant (Total number of intrusions: *F*_(6,141)_ = 1.98, *p* = 0.072, R^2^ = 0.08, BF^01^ = 8.82; IIC: *F*_(6,141)_ = 0.99, *p* = 0.44, R^2^ = 0.04, BF^01^ = 92.58). Therefore, there was no evidence that intentional intrusion control differed between the groups, nor was this moderated by executive functioning.

### Exploratory analysis

#### Dispositional mindfulness enhances intrusion control and is moderated by baseline inhibition

As dispositional mindfulness increased for both groups, we reran the analyses for the main hypothesis using post-training scores of the MAAS as the predictor variable. For the total number of intrusions, the overall model was significant (*F*_(6,141)_ = 3.011, *p* = 0.008, R^2^ = 0.11, BF^10^ = 1.23). Dispositional mindfulness alone did not predict the total number of intrusions (MAAS: *b* = − 0.59, *t* = − 0.84, *p* = 0.40, CI [− 1.99, 0.81], BF^01^ = 2.59). Inhibition moderated the effect of dispositional mindfulness on the total number of intrusions (MAAS*Stroop: *b* = − 0.003, *t* = 2.66, *p* = 0.009, CI [0.001, 0.006], BF^10^ = 7.39). In contrast, there was no moderating effect of working memory (MAAS*OSPAN: *b* = − 0.008, *t* = − 0.56, *p* = 0.58, CI [− 0.037, 0.021], BF^01^ = 2.42). Furthermore, compliance scores did not influence the model (*b* = 2.17, *t* = 0.99, *p* = 0.32, CI [− 2.14, 6.49], BF^01^ = 2.32).

**Figure 4:**
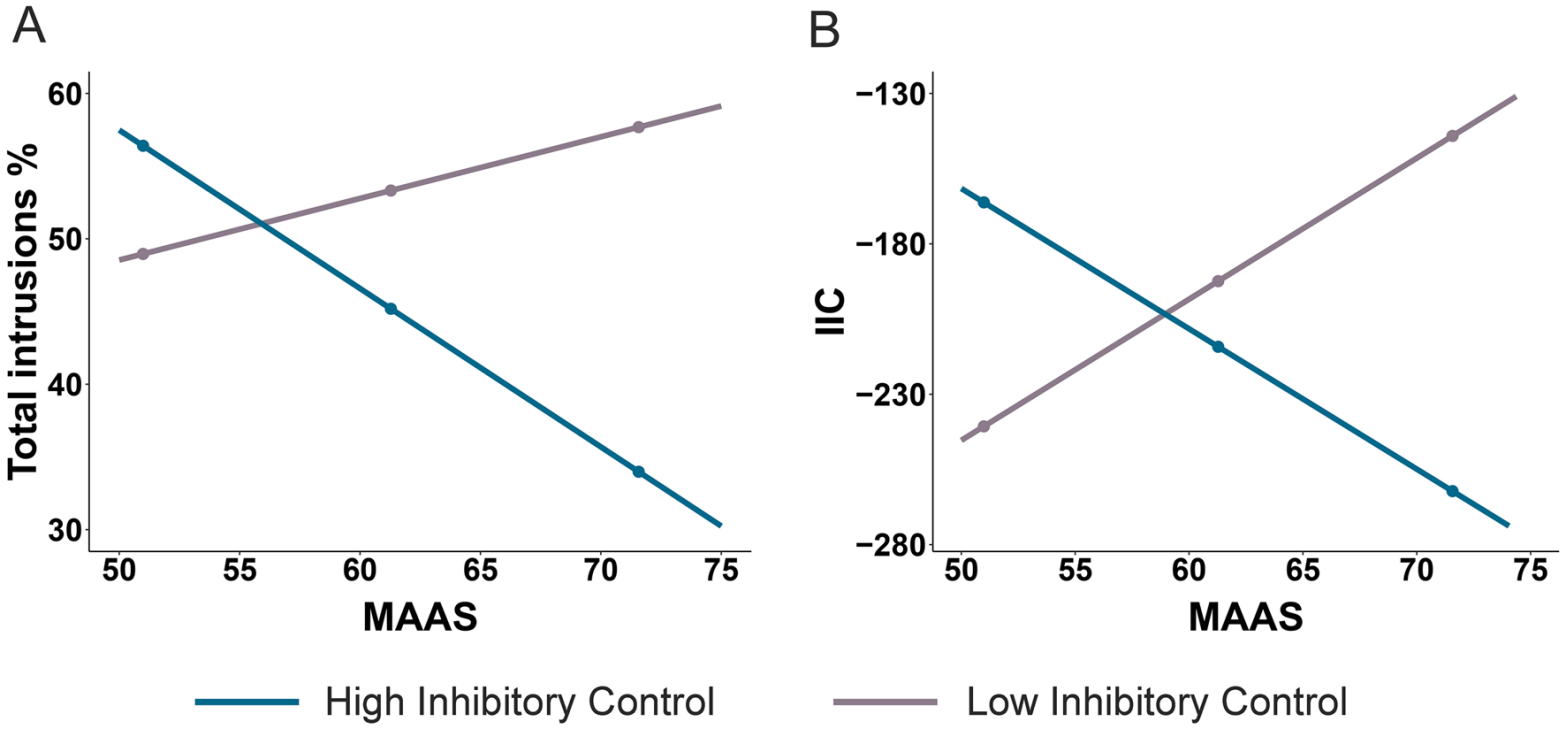
The effect of dispositional mindfulness on intrusions is moderated by baseline inhibition. The graphs present a visualisation of the interaction effects between inhibition (Stroop) and post-test scores on the MAAS as predictors of total intrusions (**A**) and intrusion control, IIC (**B**). Simple slope analyses on the conditional effects of dispositional mindfulness (MAAS) are significant at high levels of Stroop for total intrusions and at both high and low levels of Stroop for IIC.

To follow up on the significant interaction between the dispositional mindfulness and inhibition, we reran the analysis after removing non-significant paths from the model. Increased dispositional mindfulness predicted fewer total intrusions (MAAS: *b* = − 1.01, *t* = − 3.28, *p* = 0.001, CI [− 1.61, − 0.40], BF^10^ = 0.39). The effect of dispositional mindfulness on the total number of intrusions is moderated by inhibition (MAAS*Stroop: *b* = − 0.004, *t* = 3.30, *p* = 0.001, CI [0.002, 0.006], BF^10^ = 33.18). To explore the interaction, further simple slope analyses on the conditional effects of dispositional mindfulness were tested at low (Stroop_(low)_: − 1SD) and high (Stroop_(high)_: + 1SD) levels of inhibition. At higher levels of inhibition, increased dispositional mindfulness predicted fewer total intrusions (Stroop_(high)_: *b* = − 1.09, *t* = − 3.35, *p* = 0.001, CI [− 1.73, − 0.45]; see Fig. 4). At lower levels of inhibition, there was no relationship between dispositional mindfulness and total intrusions (Stroop_(low)_: *b* = 0.42, *t* = 1.40, *p* = 0.17, CI [− 0.18, 1.02]).

Next, we performed the same analysis to predict the ability to control intrusions using the IIC. The overall model proved significant (*F*_(6,141)_ = 2.75, *p* = 0.015, R^2^ = 0.12, BF^10^ = 0.68). Dispositional mindfulness alone was not found to predict intrusion control (MAAS: *b* = -1.35, *t* = − 0.34, *p* = 0.73, CI [− 9.27, 6.56], BF^01^ = 5.53). Inhibition moderated the effect of dispositional mindfulness on intrusion control (MAAS*Stroop: *b* = − 0.021, *t* = 2.94, *p* = 0.004, CI [0.007, 0.035], BF^10^ = 13.97). There was no moderation of working memory (MAAS*OSPAN: *b* = − 0.056, *t* = 1.42, *p* = 0.16, CI [− 6.91, 41.84], BF^01^ = 2.22). Furthermore, compliance scores did not influence the model (*b* = 17.46, *t* = 1.41, *p* = 0.16, CI [-6.91, 41.84], BF^01^ = 1.49).

To follow-up on the significant interaction between the dispositional mindfulness and inhibition, we reran the analysis after removing non-significant paths from the model. Increased dispositional mindfulness predicted an increased ability to downregulate intrusions (MAAS: *b* = − 4.15, *t* = − 2.38, *p* = 0.019, CI [− 7.59, − 0.71], BF^10^ = 0.18). Furthermore, the effect of dispositional mindfulness on intrusion control is moderated by inhibition (MAAS*Stroop: *b* = − 0.023, *t* = 3.60, *p* < 0.001, CI [0.011, 0.036], BF^10^ = 78.74). Simple slope analyses on the conditional effects of dispositional mindfulness at low and high levels of Stroop revealed that both increased and decreased inhibition significantly contributed to the effect of dispositional mindfulness on IIC (see Fig. 4). At higher levels of inhibition, increased dispositional mindfulness increased the ability to downregulate intrusions (Stroop_(high)_: *b* = − 4.67, *t* = − 2.53, *p* = 0.013, CI [− 8.31, − 1.02]). In contrast, at lower levels of inhibition, increased dispositional mindfulness decreased the ability to downregulate intrusions (Stroop_(low)_: *b* = 4.69, t_(148)_ = 2.73, *p* = 0.007, CI [1.29, 8.10]).

### The influence of task compliance and subjective suppression ability on intentional suppression

As an exploratory analysis, we tested whether we could replicate the results of Liu et al.^47^, which found that SIF diminishes with reduced compliance. To analyse the full range of compliance scores, the sample included participants with a score > 3 who were previously excluded (*n* = 24; Note: one additional participant was excluded from this sample due to no audio recording for the learning phase. As such, independent scoring could not be determined). Contrasting the original findings, compliance was not significantly associated with SIF (*r*_(169)_ = 0.13, *p* = 0.085, BF^01^ = 2.38). Furthermore, compliance was not associated with the total number of intrusions (*r*_(171)_ = 0.025, *p* = 0.75, BF^01^ = 9.93) or IIC (*r*_(171)_ = 0.067, *p* = 0.38, BF^01^ = 7.14).

We also explored whether subjective suppression ability during the task influenced measures of intrusions. Subjective suppression ability was negatively correlated with both total intrusions (*r*_(148)_ = − 0.66 *p* < 0.001, BF^10^ > 100) and IIC (*r*_(148)_ = − 0.42 *p* < 0.001, BF^10^ > 100), demonstrating that increased subjective suppression ability during No-Think trials was associated with enhanced intrusion control. Subjective suppression ability during phase 2 was not found to be associated with subsequent SIF (*r*_(146)_ = 0.059, *p* = 0.49, BF^01^ = 7.56).

## Discussion

This experiment investigated whether practicing mindfulness enhanced the ability to control intrusive memories. Furthermore, we investigated whether increased executive functioning at baseline moderated this effect, which would lead to greater enhancement in intrusion control. To test this, participants first completed the Stroop and Operation span task to measure executive functioning. Next, participants underwent either a 10 day mindfulness training from home or listened to podcasts. On the final day, participants returned to the lab and completed the Think/No-Think task to measure intrusions. Intrusions for No-Think trials were quantified as both the total frequency and the IIC, in order to provide insight into both the quantity and quality of intrusion control. Our planned analyses compared participants that took part in an online mindfulness training to an active control group. Contrary to our hypothesis and results of the pilot data, both groups increased equally in dispositional mindfulness between baseline and post-test. As such, exploratory analyses were performed with post-training dispositional mindfulness across the whole sample. Increased dispositional mindfulness resulted in fewer intrusions and enhanced the ability to downregulate memory intrusions over time. Furthermore, inhibition at baseline moderated this effect.

As originally proposed by Levy and Anderson^25^, individual differences in the ability to regulate intrusive memories may be influenced by pre-existing differences in executive control. As such, we assessed baseline measures of inhibition via the Stroop task and working memory via the OSPAN. Whereas working memory was not found to have an effect, high levels of inhibition at baseline moderated the effect of dispositional mindfulness on both the total intrusions and IIC, resulting in fewer intrusions and an enhanced ability to downregulate intrusions. In contrast, at lower levels of inhibition, increased dispositional mindfulness decreased the ability to downregulate intrusions. Previous research has shown that individuals with a higher working memory capacity on the OSPAN show greater inhibition on the Stroop task^54^. Although argued to be close in relation, these are considered separate constructs that contribute to a larger body of executive functions^26,27^. The current results support this, as the findings suggest that the constructs of inhibition and working memory differ in their influence on memory control. Furthermore, our findings support the theory for the role of inhibition as the underlying mechanism behind intentional memory control^55^.

Results demonstrated that, from day 1 to day 10, all participants increased in dispositional mindfulness. There is debate over what constitutes an appropriate control group for a mindfulness study^38,56^. Our choice for the active control task was similar to previous studies that have also used audio-based tasks^35,57^. The podcasts were matched to the intervention group on several factors (i.e. Headspace produced content and matched on time duration). The rationale behind choosing the control task was that, although the content was mindfulness based, participants were not actively practicing mindfulness techniques. In doing so, we aimed to create a sham condition that could give insight into the placebo effect discussed in mindfulness research^58^. Overall, participants reported at the start of the study that they believed mindfulness had the potential to be beneficial. As such, the sample could have been biased to the belief that they would gain something from their online tasks. On the other hand, it could be that the content of the active control task increased dispositional mindfulness on its own merit. The content could have resulted in reflective thinking or acted as affirmations, which are terms that have both contributed to defining the concept of mindfulness^59^.

To check adherence to task instructions, we used the compliance questionnaire employed by Liu et al.^47^. Furthermore, we used their suggested cut-off criteria to exclude participants who had a total score of higher than 3, as scores higher than this were found to diminish the SIF effect. In the exploratory analysis, we did not replicate the original finding that compliance scores are negatively associated with SIF. We recommend that researchers first check whether compliance scores are found to influence measures of suppression induced forgetting. If so, the proposed cut-off of > 3 should be imposed during analysis, rather than during data collection. It should also be noted that more recent recommendations suggest to use a cut-off > 4^51^. Alternatively, compliance scores could be added as a covariate during analysis. Participants who completed the mindfulness training demonstrated higher compliance to task instructions compared to controls. Importantly, this was not found to influence our main hypothesis. Furthermore, due to the cut-off score imposed, all participants can be considered to have high compliance with task instructions. A key factor to ensure compliance and task understanding is delivery of instructions during the practice phases for the T/NT task. Here, the suppression strategies of participants are discussed so the experimenter can check adherence to instructions. Our compliance scores were higher compared to the original study. This could have occurred due to our framing of how to answer the questionnaire (see Methods, Task motivation). Although, how the questions should be introduced were not described in the original paper. For future research, we advise including more detail on what instructions are given to participants and what is verbally discussed (see participant instructions and experimenter script on OSF). Streamlining methodology between studies is of critical importance to produce replicable results.

In addition to compliance, we also explored the effect of subjective suppression ability. We found that increased subjective ability during intentional suppression was associated with an enhanced ability to downregulate intrusions and with fewer intrusions reported overall. No association was observed between subjective suppression ability and subsequent SIF. These scales quantified participants perceived difficulty and success in suppressing, as well as motivation. Future research should take subjective suppression ability into account when interpreting their results, as it may provide further insight into factors that influence the ability to suppress. In the current study, suppression ability was assessed for neutral stimuli. Fewer intrusions have previously been reported for unpleasant (i.e. stimuli that evoked disgust) compared to neutral stimuli^60^. It could be the case that participants are more motivated to suppress negative stimuli, and thus are more effective in controlling intrusions. Future studies should build on the current findings by investigating whether dispositional mindfulness influences how individuals control intrusions for negative stimuli and the role of motivation in this process. Furthermore, future research could look at improving the method of capturing intrusions. It could be the case that asking participants how often the target comes to mind acts as an unwanted reminder of the target that the participant is aiming to suppress. One method could be to have the participant freely press a button if and when a target word intrudes during the presentation of a cue, rather than ask the question directly at the end. It would be of interest see if an alternative method of intrusion ratings produces comparable SIF effects to the traditional method employed in the current study (see^49^ for methodological considerations in measuring intrusions in T/NT tasks).

It should be noted that this psychologically healthy sample consisted largely of psychology students, all of a similar age. To advance, the findings should be replicated to see if this effect translates in more diverse samples, including patient populations. Deficits in intentional memory control are observed in numerous clinical disorders that are characterised by the occurrence of unwanted thoughts^4^. These deficits could act as the mechanism that underlie central symptomatology such as ruminative thinking in depression^61,62^, worrying in anxiety^63,64^ or the persistence of traumatic memories in PTSD^37,65^. Mindfulness based therapies have been shown to be effective treatments for mood and anxiety disorders^66^. Targeting specific symptoms, as opposed to disorders as a whole, may improve treatment outcomes^67^ and give insight into specific mechanisms that benefit from mindfulness.

The current results demonstrate that increased dispositional mindfulness results in fewer intrusions and enhances the ability to downregulate intrusions. As this effect was observed in both groups, we cannot provide evidence for which specific factors caused this increase in mindfulness. Furthermore, the effect of mindfulness on intrusions was dependent on baseline inhibition. These findings highlight mindfulness as a promising avenue to enhance the ability to control unwanted thoughts and further demonstrate the role of executive functioning in this effect.

## Data Availability

Pilot data is available at https://osf.io/qjk3d/. Raw data and materials from stage 2 are available at OSF.

## Code availability

Task scripts and pilot analysis from stage 1 are available at https://osf.io/qjk3d. Open source code for stage 2 analyses are available at OSF.
